# Burnout syndrome, extracurricular activities and social support among Brazilian internship medical students: a cross-sectional analysis

**DOI:** 10.1186/s12909-020-01998-6

**Published:** 2020-03-18

**Authors:** Maria Carolina Pedro Fontana, Igor Prado Generoso, Alexandre Sizilio, Danielle Bivanco-Lima

**Affiliations:** 1 Faculdade de Ciências Médicas da Santa Casa de São Paulo, São Paulo, Brazil; 2 Universidade Cidade de São Paulo (UNICID), São Paulo, Brazil; 3 Public Health Department of Faculdade de Ciências Médicas da Santa Casa de São Paulo, Rua Doutor Cesario Mota Júnior, 61, 5° andar, sala 3, São Paulo, SP 01221-906 Brazil

**Keywords:** Burnout, Medical students, Social support, Educational activities, Burnout, Estudantes de Medicina, Suporte Social, Atividades Educacionais

## Abstract

**Background:**

Burnout syndrome (BS) is highly prevalent among medical students and is associated with lower empathy and worsening of medical students ´ mental health. The aim of our study was to identify prevalence of BS during internship and its association with self-rated social support and participation in extracurricular activities in one medical school in Brazil.

**Methods:**

This was a cross-sectional study conducted in 2015, with 121 medical students on internship (56% response rate). They were evaluated using the Maslach Burnout Inventory – Human Services (MBI) and assessed about socio demographic data, social support and extracurricular activities.

**Results:**

The overall BS prevalence was 57.5% among medical interns. High emotional exhaustion was present in 33.1% (*N* = 38) of interns, high depersonalization was observed in 45.7% (*N* = 58) and 36.2% of participants (*N* = 46) had low personal accomplishment. Individuals with participation in community services had lower frequency of depersonalization (prevalence ratio 0.61 CI95% 0.42–0.88). BS was not associated with different types of extracurricular activities and no association was found among BS and the behaviour of seeking social support.

**Conclusions:**

We found high prevalence of BS in medical interns, however the behaviour of seeking social support had no association with BS. The interns participating in community activities had lower frequency of high depersonalization.

## Resumo

**Introdução:**

A síndrome de Burnout (SB) apresenta alta prevalência entre os estudantes de medicina e está associada a menor empatia e pior saúde mental. O objetivo deste estudo foi identificar a prevalência da SB durante o internato e sua associação com suporte social auto-relatado e atividades extracurriculares em uma faculdade de medicina brasileira.

**Métodos:**

Estudo transversal conduzido em 2015, com 121 internos (taxa de resposta de 56%). Foram avaliados através da escala validada Maslach Burnout Inventory - Human Services (MBI) e questionário sociodemográfico que incluiu informações sobre suporte social e participação em atividades extracurriculares.

**Resultados:**

A prevalência total de SB foi 57,5% entre os estudantes. Alta exaustão emocional foi observada em 33,1% (*N* = 38) dos alunos, alta despersonalização foi observada em 45,7% (*N* = 58) e 36,2% dos participantes (*N* = 46) apresentou reduzida realização pessoal. Indivíduos que participaram de serviços comunitários apresentaram menor frequência de alta despersonalização (razão de prevalência 0,61, intervalo de confiança 95% 0,42-0,88). A SB não apresentou associação com demais grupos de atividades extracurriculares ou com grupos de suporte social.

**Conclusões:**

Foi observada alta prevalência de SB em internos de medicina, no entanto o comportamento de procura de suporte social não se associou com SB. Os internos que apresentavam participação em atividades extracurriculares comunitárias apresentavam menor frequência de alta despersonalização.

## Background

Burnout syndrome (BS) is highly prevalent among medical students and apparently it was first described by Herbert Freudenberger, a psychologist working with volunteers managing individuals with drug disorders [1–4]. However, there is evidence that 3 years before, air traffic controllers in United States used the term burnout for exhaustion related to work demands, resulting in decline of production [4].

BS is characterized by a model of emotional suffering regarding work demands and includes three categories of symptoms: emotional exhaustion (EE), depersonalization (DP) and reduced personal accomplishment (PA). EE is described as feeling emotionally depleted and include symptoms of fatigue, low energy and increased sensibility to external stressors. DP is described as negative feelings and perceptions about the patients, including the attempt to distance oneself from patients, resulting in cynicism, lower empathy and indifference. Low PA is characterized by the negative self-evaluation, loss of satisfaction, motivation and efficiency on work related activities [5, 6].

The 11th Revision of the International Classification of Diseases (ICD-11) describes burnout as a specifically occupational syndrome. It is described as chronic workplace stress not successfully managed, with three dimensions: feelings of energy depletion or exhaustion, increased mental distance from one’s job, or feelings of negativism or cynicism related to one’s job, and reduced professional efficacy [7].

Burnout syndrome has negative consequences in medical students´ health and is associated with suicidal ideation and psychiatric disorders [2]. Dyrbye et. al. evaluated 545 medical students and found 45% prevalence of BS using Maslach Burnout Inventory (MBI), 35% with high EE, 26% with high DP and 31% with low PA. They also found that 56% had positive depressive disorder screening, 22% had high risk alcohol use and 14.7% had binge drinking. Negative life events (divorce, personal illness, illness in a close family member and/or death) were associated with higher risk of BS (*p* = 0.016) [8]. Psychological distress (including BS) can also impact negatively on academic performance, and adversely affect the learning process and development of competences during medical training [3, 9–11]. BS is associated with lower empathy and worse professional climate among medical students. Brazeau et al. evaluated 127 medical students and found that individuals with higher scores of EE, DP and PA reported worse perception of professional climate (*p* < 0.01) and lower score of self-rated empathy [12].

BS is expected to be seen in up to half of all medical students during their undergraduate period, with the risk of persisting after graduation. A recent review of BS in medical students and residents using articles from 1990 to 2015 found that the prevalence of BS in medical students ranges from 45 to 56% among different populations, with a range of 35 to 45% of high EE and 26 to 38% of high DP [3]. A review of mental health problems in Brazilian medical students showed only three studies on this population with a pooled prevalence of 13.1% (confidence interval 95% 10.2–16.4) [13]. However, this study was conducted in initial years of the course and used different way to analyse data about BS. A Brazilian study in medical interns showed a 63.2% prevalence of high EE, 53.8% prevalence of high DP and 50.9% prevalence of low PA [14].

There is some evidence about protective factors of BS in medical students, as positive life events (marriage, birth or adoption), social support, extracurricular activities and physical activity [8, 15–17]. A cross-sectional Spanish study with health professionals found that individuals with higher and moderate self-esteem are associated with higher social support and empathy and lower frequency of BS [18]. Thompson et. al. found that medical students with lower perceived social support had higher rates of EE (*p* = 0.04) [15]. A good perceived social support can buffer stress related to academic workload or help the students to cope [19, 20] and seeking social support is seen as a functional strategy to cope with BS [21].

The association among BS and extracurricular activities is controversial, although there is evidence pointing to the protective effect of exercise [17, 22, 23]. Fares et. al evaluated medical students ´ extracurricular activities and found no association with BS, except for a protective factor with music related activities (*p* = 0.045) [22]. Almalki also found no association among extracurricular activities and BS (*p* = 0.33) [23]. Shadid et. al. did not observed an association between BS and extracurricular activities, however medical students that had none of the extracurricular activities evaluated had higher risk of high stress (Odds Ratio (OR) 1.89 CI 95% 1.23–2.91) [24]. The extracurricular activities could be viewed as an expression of autonomy, increasing students ´ motivation.

Medical undergraduate in Brazil is a six-year duration course because of the absence of pre-medical training on our local educational context. The internship encompasses the last 2 years of medical training in Brazil, with mainly workplace training (primary care, ambulatories and hospital settings). The aim of our study was to identify prevalence of BS during internship and its association with extracurricular activities and social support in one medical school in São Paulo, Brazil.

## Methods

This was a cross-sectional study conducted at Faculdade de Ciências Médicas da Santa Casa de São Paulo, a traditional medical school from São Paulo (Brazil) during 2015. The population of interest were internship students (including medical students of 5th and 6th years). The eligibility criteria were to be an internship student during 2015. All the medical students attending internship were invited to participate. The participants were at different rotations (practice in Surgery, Pediatrics, Primary Care, Gynecology, Internal Medicine, Emergency, Orthopedics or Infectology). The students were contacted between July and November of 2015. The population of the internship students at Faculdade de Ciências Médicas da Santa Casa de São Paulo was composed by 216 individuals during 2015.

The students were evaluated regarding socio demographic data, extracurricular activities, self-rated social support and burnout syndrome. It was used the Maslach Burnout Inventory – Human Services for assessment of burnout syndrome, a validated instrument, with three subscales: Emotional Exhaustion; Depersonalization; Personal Accomplishment [5].

For the purposes of this study, the overall prevalence of BS was assessed by the presence of a high score on one or both the subscales EE and DP. High EE was defined by a subscale score on MBI of 27 or higher. DP was defined by a subscale score on MBI of 10 or higher. Low PA was defined as a subscale score of 34 or lower, but it is not included to compose overall prevalence of BS [5, 25–27]..

The extracurricular activities were combined in 4 categories: academic programs (which consists of involvement in research or being a part of a study group or participating in courses and academic leagues); political involvement (being a member from the student union or student representative at faculty and college administration meetings); volunteer and community services (to participate in activities or other groups that provide volunteer work) and athletic activities (member from the athletic association or athletic band).

Social support was evaluated by self-reported behaviour of seeking emotional support and with whom they would share their feelings and worries (family, friends outside medical school, friends from their rotation, friends from their school but from different years/rotations and/or with their romantic partner). Sociodemographic data were evaluated such as age, monthly household income (counted by minimum wages, equivalent to R$788,00 or U$252,56 by the time of data collection), parents ´ educational level, and ownership of research grants or scholarships.

### Statistical analysis

The analysis included descriptive summary statistics (means and proportions) for demographic data, associated variables and prevalence of BS, EE and DP. Differences among individuals with overall BS, high EE and high DP were evaluated using Chi-square test (Fischer exact test, when indicated). The comparisons among categorical and continuous variables used ANOVA one-way test (for age). The prevalence ratio was calculated with Poisson Test (indicated when prevalence of independent variable higher than 20%) and its confidence intervals. The tests are two sided and were considered a type I error rate of 0.05 (alpha error < 0.05). Data were analysed with STATA/SE software version 13.1.

### Ethical considerations

The study was submitted to the Ethics Board of Irmandade da Santa Casa de São Paulo and approved. The study protocol was approved under number CAAE 31151315.5.0000.5479 in March, 2015. All participants that were invited and agreed to participate in the research, signed the informed written consent.

## Results

A total of 121 internship medical students agreed to participate and fully answered the questionnaires (56% participation rate). The participants were 68 (56.2%) male students and 53 (43.8%) female students, with similar mean age (25 years; *p* = 0.08) and monthly household income (*p* = 0.17). The sample was balanced among first and second year of internship individuals (45.5% of 1th year interns and 54.5% of 2th year interns; *p* = 0.44). Among men, 31.8% had research grants and the rate was 39.6% among women (*p* = 0.376). There were gender differences regarding scholarships, with higher frequency for female (54.7%) over male students (35.8%; *p* = 0.038). For participation on extracurricular activities, both genders had high frequency on academic programs (94.3% of students, *p* = 0.105). Concerning social support reported by the students, 88.7% of women reported to share their problems with their family, while 72.1% of men reported the same behaviour (*p* = 0.025) (Table 1). Male students also report lower frequency of seeking support from students in other rotations (*p* = 0.044). The prevalence of high emotional exhaustion was found in 33.1% (*N* = 38) of the participants, 45.7% (*N* = 58) had high depersonalization and 36.2% (*N* = 46) had low personal accomplishment (Table 1).

**Table 1:** Socio-demographic characteristics, extracurricular activities, social support and burnout syndrome prevalence of interns according to gender, FSMSCSP, São Paulo, 2015

	Male Gender ***N*** **= 68**	Female Gender ***N*** **= 53**	Total ***N*** **= 121**	*p*-value
Mean age in years (standard deviation)	25.2 (2.5)	25.3 (1.9)	25.3 (2.3)	0.392
Year in medical internship (%)				0.442
1st	33 (48.5)	22 (41.5)	55 (45.5)	
2nd	35 (51.5)	31 (58.5)	66 (54.5)	
Monthly household income (%)				0.174
15 minimum wages or lower	26 (39.4)	27 (51.9)	53 (43.8)	
Higher than 15 minimum wages	40 (60.6)	25 (48.1)	65 (53.7)	
Parents with university education (%)				
Father	53 (77.9)	45 (84.9)	98 (80.9)	0.333
Mother	49 (74.2)	43 (81.1)	92 (77.3)	0.372
Research grant (%)				0.376
Yes	21 (31.8)	21 (39.6)	42 (35.3)	
No	45 (68.2)	32 (60.4)	77 (64.7)	
Scholarship (%)				0.038
Yes	24 (35.8)	29 (54.7)	53 (44.2)	
No	43 (64.2)	24 (45.3)	67 (55.8)	
Extracurricular activities (%)				
Academic programs	62 (96.8)	52 (98.1)	114 (94.3)	0.105
Political involvement	21 (36.2)	20 (43.4)	41 (33.9)	0.472
Community services	58 (90.6)	45 (86.5)	103 (85.1)	0.952
Athletic activities	32 (54.2)	26 (53)	58 (47.9)	0.825
Self reported social support (%)				
Family	49 (72.1)	47 (88.7)	96 (79.3)	0.025
Medical students in the same rotation	63 (92.7)	51 (96.2)	114 (94.2)	0.403
Medical students in other rotations	47 (69.1)	45 (84.9)	92 (76.0)	0.044
Friends from outside medical school	44 (64.7)	39 (73.6)	83 (68.6)	0.296
Partner	47 (69.1)	42 (80.8)	89 (74.2)	0.148
Overall Burnout Syndrome (%)	35 (51.5)	32 (60.4)	67 (55.4)	0.328
High emotional exhaustion	18 (27.7)	20 (40)	38 (33)	0.164
High depersonalization	31 (45.6)	21 (39.6)	52 (43)	0.511
Low personal accomplishment	28 (41.2)	18 (34)	46 (38)	0.417

The ownership of research grant or scholarship had no association with EE, DP or low PA. The different categories of extracurricular activities didn’t have association with EE and low PA. Students with participation in community services had lower prevalence of high depersonalization (prevalence ratio (PR) 0.61, confidence interval 95% (CI 95%) 0.42–0.88) (Table 2). None of the characteristics of self-reported social support is associated with BS subscales (Table 2).

**Table 2:** Socio demographic characteristics, extracurricular activities and social support of interns, according to Burnout Syndrome Subscales, FCMSCSP - São Paulo, Brazil, 2015

	High Emotional Exhaustion			High Depersonalization			Low Personal Accomplishment		
	YES	NO	**PR (CI 95%)**	YES	NO	**PR (CI 95%)**	YES	NO	**PR (CI 95%)**
	**N (%)**	**N (%)**		**N (%)**	**N (%)**		***N* = (%)**	**N (%)**	
Overall	38 (33.1)	77 (66.9)	–	58 (45.7)	69 (54.3)	–	46 (36.2)	81 (63.8)	–
Year in medical internship									
1st	14 (28)	36 (72)	1	22 (40)	33 (60)	1	21 (38.2)	34 (61.8)	1
2nd	24 (36.9)	41 (63.1)	1.31 (0.76–2.28)	30 (45.5)	36 (54.5)	1.13 (0.74–1.73)	25 (37.9)	41 (62.1)	0.99 (0.63–1.57)
Monthly household income									
15 minimum wages or lower	17 (34)	33 (66)	1	20 (37.7)	33 (62.3)	1	17 (32.1)	36 (67.9)	1
Higher than 15 minimum wages	20 (32.3)	42 (67.7)	0.94 (0.55–1.61)	30 (46.1)	35 (53.9)	1.22 (0.79–1.89)	28 (43.1)	37 (56.9)	1.34 (0.83–2.18)
Research grant									
Yes	15 (38.5)	24 (61.5)	1.23 (0.73–2.09)	23 (54.8)	19 (45.2)	1.45 (0.97–2.16)	13 (30.9)	29 (69.1)	0.74 (0.44–1.26)
No	23 (31.1)	51 (68.9)	1	29 (37.7)	48 (62.3)	1	32 (41.6)	45 (58.4)	1
Scholarship									
Yes	20 (39.2)	31 (60.8)	1.45 (0.85–2.47)	22 (41.5)	31 (58.5)	0.95 (0.62–1.46)	20 (37.7)	33 (62.3)	1.01 (0.63–1.61)
No	17 (27)	46 (73)	1	29 (43.3)	38 (56.7)	1	25 (37.3)	42 (62.7)	1
Extracurricular activities									
Ever been involved in academic activities	36 (33)	73 (67)	0.99 (0.31–3.19)	51 (44.7)	63 (55.3)	0.83 (0.48–1.43)	44 (38.6)	70 (61.4)	2.5 (0.68–9.2)
Never been involved in academic activities	2 (33.3)	4 (66.7)	1	7 (53.8)	6 (46.2)	1	2 (15.4)	11 (84.6)	1
Ever been involved in political activities	10 (41.7)	14 (58.3)	1.35 (0.76–2.38)	8 (30.8)	18 (69.2)	0.62 (0.33–1.14)	6 (23.1)	20 (76.9)	0.58 (0.28–1.23)
Never been involved in political activities	28 (30.8)	63 (69.2)	1	50 (49.5)	51 (50.5)	1	40 (39.6)	61 (60.4)	1
Ever been involved with community services	32 (32)	68 (68)	0.8 (0.40–1.59)	42 (40.8)	61 (59.2)	0.61 (0.42–0.88)	40 (38.8)	63 (61.2)	1.55 (0.74–3.24)
Never been involved with community services	6 (40)	9 (60)	1	16 (66.7)	8 (33.3)	1	6 (25)	18 (75)	1
Ever been involved with athletic activities	14 (31.1)	31 (68.9)	0.9 (0.52–1.56)	19 (40.4)	28 (59.6)	0.82 (0.54–1.25)	17 (36.2)	30 (63.8)	0.99 (0.61–1.61)
Never been involved with athletic service	24 (34.3)	46 (65.7)	1	39 (48.7)	41 (51.3)	1	29 (36.2)	51 (63.8)	1
Self reported social support									
Family									
Yes	29 (32.2)	61 (67.8)	0.89 (0.49–1.64)	42 (43.7)	54 (56.3)	1.09 (0.64–1.86)	38 (39.6)	58 (30.4)	1.23 (0.66–2.31)
No	9 (36)	16 (64)	1	10 (40)	15 (60)	1	8 (32)	17 (68)	1
Medical students in the same rotation									
Yes	36 (33.3)	72 (66.7)	1.17 (0.34–3.9)	49 (43)	65 (57)	1.00 (0.41–2.42)	45 (39.5)	69 (60.5)	2.76 (0.44–17.3)
No	2 (28.6)	5 (71.4)	1	3 (42.9)	4 (57.1)	1	1 (14.3)	6 (85.7)	1
Medical students in other rotations									
Yes	30 (34.1)	58 (65.9)	1.15 (0.59–2.21)	39 (42.4)	53 (57.6)	0.94 (0.59–1.51)	37 (40.2)	55 (59.8)	1.29 (0.71–2.36)
No	8 (29.6)	19 (70.4)	1	13 (44.8)	16 (55.2)	1	9 (31)	20 (69)	1
Friends from outside medical school									
Yes	27 (33.8)	53 (66.2)	1.07 (0.60–1.92)	38 (45.2)	46 (54.8)	1.19 (0.74–1.93)	32 (38.1)	52 (61.9)	1 (0.61–1.65)
No	11 (31.4)	24 (68.6)	1	14 (37.8)	23 (62.2)	1	14 (37.8)	23 (62.2)	1
Partner									
Yes	28 (31.5)	61 (68.5)	0.81 (0.45–1.45)	35 (38.9)	55 (61.1)	0.72 (0.48–1.11)	30 (33.3)	60 (66.7)	0.66 (0.41–1.06)
No	10 (38.5)	16 (61.5)	1	16 (53.3)	14 (46.7)	1	15 (50)	15 (50)	1

The burnout syndrome was defined as the presence of high emotional exhaustion and/or high depersonalization. The prevalence of BS does not differ among first and second year’s interns (PR 1.39 CI 95% 0.67–2.86). Neither household income, ownership of research grant, or scholarship was associated with overall BS prevalence. The involvement in extracurricular activities has no association with the overall BS prevalence, neither any subtype of self-related social support (Table 3).

**Table 3:** Socio demographic characteristics, extracurricular activities and social support of interns, according to the presence of Burnout Syndrome, FCMSCSP - São Paulo, Brazil, 2015

	Burnout Syndrome		
	YES (*n* = 73)	NO (*n* = 54)	**PR (CI95%)**
	**N (%)**	**N (%)**	
Year in medical internship			
1st	28 (50.9)	27 (49.1)	1
2nd	39 (59.1)	27 (40.9)	1.39 (0.67–2.86)
Monthly household income			
15 minimum wages or lower	30 (56.6)	23 (43.4)	1
Higher than 15 minimum wages	38 (58.5)	27 (41.5)	1.03 (0.49–2.13)
Research grant			
Yes	28 (66.7)	14 (33.3)	1.94 (0.89–4.25)
No	39 (50.7)	38 (49.4)	1
Scholarship			
Yes	33 (62.3)	20 (37.7)	1.70 (0.81–3.53)
No	33 (49.3)	34 (50.8)	1
Extracurricular activities			
Ever been involved in academic activities	65 (57.1)	49 (42.9)	0.83 (0.25–2.69)
Never been involved in academic activities	8 (61.5)	5 (38.5)	1
Ever been involved in political activities	13 (50)	13 (50)	0.68 (0.28–1.62)
Never been involved in political activities	60 (59.4)	41 (40.6)	1
Ever been involved with community service	55 (53.4)	48 (46.6)	0.38 (0.14–1.04)
Never been involved with community service	18 (75)	6 (25)	1
Ever been involved with athletic activities	24 (51.1)	23 (48.9)	0.66 (0.31–1.36)
Never been involved with athletic service	49 (61.3)	31 (38.7)	1
Self reported social support			
Family			
Yes	54 (56.3)	42 (43.7)	1.18 (0.49–2.86)
No	13 (52)	12 (48)	1
Medical students in the same rotation			
Yes	64 (56.1)	50 (43.9)	1.71 (0.36–7.98)
No	3 (43.9)	4 (57.1)	1
Medical students in other rotations			
Yes	53 (57.6)	39 (43.4)	1.45 (0.63–3.36)
No	14 (48.3)	15 (51.7)	1
Friends from outside medical school			
Yes	49 (58.3)	35 (41.7)	1.47 (0.67–3.21)
No	18 (48.7)	19 (51.3)	1
Partner			
Yes	49 (54.4)	41 (45.6)	0.91 (0.39–2.10)
No	17 (56.7)	13 (43.3)	1

## Discussion

This study showed high prevalence of burnout syndrome among medical interns, affecting more than half of the participants (57.5%). The prevalence of high emotional exhaustion was 33.1% (*N* = 38), 45.7% (*N* = 58) of high depersonalization and 36.2% (*N* = 46) of low personal accomplishment. Students with participation in community services as an extracurricular activity had lower prevalence of high depersonalization (PR 0.61, CI 95% 0.42–0.88).

These results are consistent with some studies that found high prevalence of BS among medical students [28, 29]. Dyrbye et. al. evidenced that medical students were more likely to have burnout syndrome and depression than the general population [30]. A recent published meta-analysis including 24 studies and 17,431 medical students showed a prevalence of 44.2% (CI 95% 33.4–55.0%) of burnout syndrome, 40.8% of high EE (CI 95% 32.8–48.9%), 35.1% of high depersonalization (CI 95% 27.2–43.0%) and 27.4% of low PA (CI 95% 20.5–34.3%) [31].

The BS prevalence is similar to other Brazilian studies analysing the interns´ population, showing high levels of emotional exhaustion and depersonalization [14, 32]. The high prevalence of BS observed is concerning since it may also interfere with quality of patients´ care, especially with high depersonalization scores [33]. A recent study in Mexico published by Miranda-Ackerman et al. evaluated 176 interns and found prevalence of 43.1% of high EE, 53.9% of high DP and 34.6% of low PA [25].

There are several factors associated with the high prevalence of BS in internship. The internship has particular challenges compared to the early years as a medical undergraduate. Some are related to closer contact with patients´ suffering and deaths, what could cause students´ suffering and internal conflicts [34]. Another challenge is the organization of educational process for the workplace training, that is very different of the previous years of medical education. There are increase in responsibilities and demands to the students, greater hours in the workplace, increasing lack of sleep, frustration of working with time constrains, lack of ideal conditions to provide care, low motivated teams and eventually unprofessional attitudes, causing moral distress among students [35, 36].

In Brazilian medical education most of internship occurs within the Unified Health System (Sistema Único de Saúde), a public health system of primary, secondary and tertiary care services. Most Brazilian medical schools have partnerships with municipalities to work within these services. These arrangements introduce the opportunity to work with poverty and vulnerable groups, what could be an extra emotional challenge for students to cope with. Another factor associated with BS in health professionals are related to work organization. Management in health services with better work processes organization could influence on health professionals´ and medical students mental health [4].

There is a conceptual model of medical student well-being integrating the idea of coping reserve of each individual that includes negative and positive inputs. Dunn and colleagues suggested that negative inputs include stress, internal conflicts, time and energy demands. And positive inputs include psychosocial support, social and healthy activities, mentorship and intellectual stimulation. These inputs interact with personality traits and temperament factors, as resilience, changing the way that each individual perceive difficulties and demands [20].

Social support could have a protective effect for BS in medical students [16, 37]. Although this study found a difference of self-rated social support sought by women, which reported to rely more on family and partners than men, this not associated with BS. This study found high frequency of the behaviour of seeking social support, as 94.2% of students reported to rely on colleagues of the same rotation, 79.3% on family, 76.0% on friends within medical school (but different rotation), 74.2% on romantic partner and 68.6% on friends outside medical school. This could be explained by the questions used to evaluate social support or the lack of evaluation of quality or sufficiency of social support. Houpy et. al. evaluated medical interns and found that, after difficult clinical events, interns have the desire to talk about it in the same day (78.1%). Those who are comfortable to talk about stress and BS had higher resilience scores [38]. Despite the fact that some evidence shows that social support is an important coping mechanism against BS, there is no evidence in the data of present study that indicates the association of seeking social support and BS among the interns.

Being a medical undergraduate is emotionally challenging and one of the proposed buffers to mental suffering is healthy extracurricular activities, as music programs, theatre or physical activity [20]. The extracurricular activities can help or worsen the balance to maintain or achieve a better mental health status. This study observed high frequencies of participation in extracurricular activities related to medical students as 94.3% participated in academic programs, 85.1% in community programs and 47.9% in athletic activities. Individuals with participation in community services had lower prevalence of high depersonalization (PR 0.61, CI 95% 0.42–0.88). There is evidence that altruism is associated with happiness, better well-being and health, even longevity [39, 40]. Although we did not find studies with the exact same results in medical education, Dyrbye et. al. surveyed 4400 students from seven American medical schools and found that as mental health improves, students had more altruistic professional beliefs, as the desire to work with underserved populations [41]. Some possible explanations are that emotional exhaustion and fatigue related to BS could reduce the willingness to work in community services as an extracurricular activity, or the depersonalization could reduce the joy related to these activities. The students with BS could also perceive the workload as critical, reducing some extracurricular activities that are perceived as less important to entrance in residency programs. However, these possibilities are still underexplored in medical education literature.

Our study had some limitations. The response rate was lower than expected, although several studies had response rates similar to our study and lower than 70% [19, 24, 25]. The cross-sectional design had limitations to infer causality. It was not used a validated tool to analyse social support and it was not asked to the students in the present study if the social support were perceived as sufficient or not. The strengths of our study are the use of a validated questionnaire to evaluate BS and the population of interns in the initial approach. The focus on protective factors of BS and its subscales (social support and extracurricular activities) is recent in medical research. More studies with longitudinal design are needed to better understand the BS causality and its association with resilience training, medical schools ´ institutional social support programmes and educational climate.

## Conclusions

The prevalence of burnout syndrome among Brazilian interns is high (57.5%). We found no association among BS and the behaviour of seeking social support among family, partners and other medical students. The prevalence ratio of high depersonalization was lower in interns that had community service extracurricular activity.

## Data Availability

The datasets generated during the current study are not publicly available due to the presence of individuals identification but are available from the corresponding author on reasonable request.

## Abbreviations

BS: Burnout syndrome
DP: Depersonalization
EE: Emotional exhaustion
ICD: International Classification of Diseases
MBI: Maslach Burnout Inventory
OR: Odds Ratio
PA: Personal Accomplishment
PR: Prevalence Ratio
